# Efficacy of bevacizumab combined with chemotherapy in the treatment of HER2-negative metastatic breast cancer: a network meta-analysis

**DOI:** 10.1186/s12885-020-6674-1

**Published:** 2020-03-04

**Authors:** Zhengwu Sun, Xiaoyan Lan, Shizhao Xu, Shen Li, Yalin Xi

**Affiliations:** 1 Department of Clinical Pharmacy, Dalian Municipal Central Hospital, Dalian, China; 2 Department of Neurology, Dalian Municipal Central Hospital, Dalian, China

**Keywords:** Bevacizumab, Chemotherapy, HER2-negative metastatic breast cancer, Network meta-analysis

## Abstract

**Background:**

It is not known what combination of bevacizumab and chemotherapy agents is the best therapeutic regimen. Comparative study results among the efficacies of bevacizumab plus chemotherapy remain controversial in patients with HER2-negative metastatic breast cancer.

**Methods:**

We searched Pubmed, Embase, and Cochrane Library Central Resister of Controlled Trials through were July 2019 for randomized controlled trials that evaluated the efficacy of bevacizumab plus chemotherapy in HER2-negative metastatic breast cancer. Data on included study characteristics, outcomes, and risk of bias were abstracted by two reviewers.

**Results:**

A total of 16 RCT studies involving 5689 patients were included. The results showed that bevacizumab (Bev) - taxanes (Tax) - capecitabine (Cap) has highest-ranking and is probably more effective for prolonging progression-free survival (PFS) than Tax, Cap, Bev-Tax and Bev-Cap, which was no convincing differences among Bev-Cap-vinorelbine, Bev-Tax-everolimus, Bev-Tax-trebananib, Bev-exemestane, Bev-Cap-cyclophosphamide in Bev-containing regimens. For overall response rate (ORR), Bev-Tax-Cap is superior to Tax, Cap and Bev-Cap, while Bev-Tax-trebananib is superior to Cap. The cumulative probability ranking showed that Bev-Tax-Cap or Bev-Tax-trebananib may have best pathological response rate in HER2-negative metastatic breast cancer.

**Conclusion:**

Our results provide moderate quality evidence that bevacizumab-taxanes-capecitabine maybe the most effective bevacizumab plus chemotherapy on PFS and ORR in HER2-negative metastatic breast cancer, however it should be also considered that bevacizumab may add toxicity to chemotherapy and whether improve overall survival (OS) or not.

## Background

As vascular endothelial growth factor (VEGF) – neutralizing antibody, bevacizumab plays a vital role in the growth and progression of neoplasm angiogenesis [1–4]. Compared with chemotherapy alone, the addition of bevacizumab to chemotherapy improves overall response rates (ORR) and procession-free survival (PFS) in patients with HER2-negative metastatic breast cancer [5, 6].

In four randomized controlled trials (RCTs), adding bevacizumab to taxanes for HER2-negative metastatic breast cancer significantly increased PFS and ORR, while combination of bevacizumab with taxanes did certainly impact on the safety profile of taxanes [7–10]. The RCT has showed that patients receiving bevacizumab-taxanes have better PFS and objective response than receiving bevacizumab-capecitabine as first-line treatment for HER2-negative metastatic breast cancer [11]. For safety profiles, bevacizumab-capecitabine has good tolerability compared with bevacizumab- taxanes [12].

Previous studies have indicated that the addition of capecitabine to taxanes and bevacizumab significantly improved PFS, OS and ORR that compared with taxanes and bevacizumab as first-line treatment strategies [13, 14]. In contrast to previous studies, other study suggested that bevacizumab plus capecitabine and taxanes did not show an improvement of PFS and safety in patients with HER2-negative metastatic breast cancer [15]. Another concern has been the addition of second-line chemotherapy agents, such as vinorelbine, everolimus and trebananib, did not improve the efficacy of bevacizumab and taxanes, while adverse events were even enhanced [16–18].

However, the best bevacizumab plus chemotherapeutic strategy is not yet available in existing clinical trials. To explore the efficacy of bevacizumab plus chemotherapy in patients with HER2-negative metastatic breast cancer (MBC), we conducted a network meta-analysis addressing the relative impact of HER2-negative MBC on PFS and ORR.

## Methods

### Search strategy

Relevant RCTs was searched in Pubmed, Embase and Cochrance library databases. Retrieval words including “bevacizumab” and “HER2 - negative Metastatic breast cancer”. In this study, subject words, free words and Boolean logic operator connection was used for retrieval without language restriction. The retrieval time was from the establishment of each database to July 2019.

### Inclusion and exclusion criteria

We included studies that i) randomized controlled clinical trials of bevacizumab based chemotherapy for HER2-negative metastatic breast cancer; ii) the baseline characteristics of patients, including age, severity of disease and underlying disease, were consistent and comparable in patients with HER2-negative metastatic breast cancer. iii) the interventions were bevacizumab based chemotherapy and conventional chemotherapy as a control.

To preserve intergroup homogeneity, we excluded that i) patients were < 18 years; ii) types of publication were case reports, reviews, commentaries and editorials, or only reported in abstract form; and iii) outcome data was incomplete or incorrect; iv) the attrition rate is more than 10%.

The above procedures of study search and selection were independently performed by two investigators (Zhengwu Sun and Yalin Xi). Study eligibility was determined by all authors’ consensus.

### Data extraction

Two investigators (Zhengwu sun and Yalin Xi) independently extracted relevant data on patient characteristics/demographics, treatment detail, outcomes, and study design, with discrepancies resolved by a third investigator. Relevant PFS and ORR were extracted for primary and secondary endpoint respectively.

### Statistical analysis

We performed direct meta-analysis for all treatment comparisons, and statistical heterogeneity tested was performed using I^2^, a value of I^2^ > 50% was considered to have substantial heterogeneity. A fixed-effects model was selected when the heterogeneity test showed I^2^ value < 50%, otherwise a random-effects model was used. The hazard ratio (HR) with its 95% CI was calculated for PFS, while the odds ratio (OR) with 95% CI was calculated for ORR. We used a bayesian random effects network meta-analysis approach to analyze the indirect data for multiple treatment comparisons. We compared the results of direct and indirect meta-analysis to determine the consistency of network meta-analysis. When it was not significant difference, we investigated consistency using consistency model, otherwise a node-splitting approach was used. All analyses were conducted in RevMan (version 3.5) and R (version 3.6.1), specifically the GeMTC package (version 0.8.2) was used for the network meta-analysis.

## Result

### Search results

**Fig. 1:**
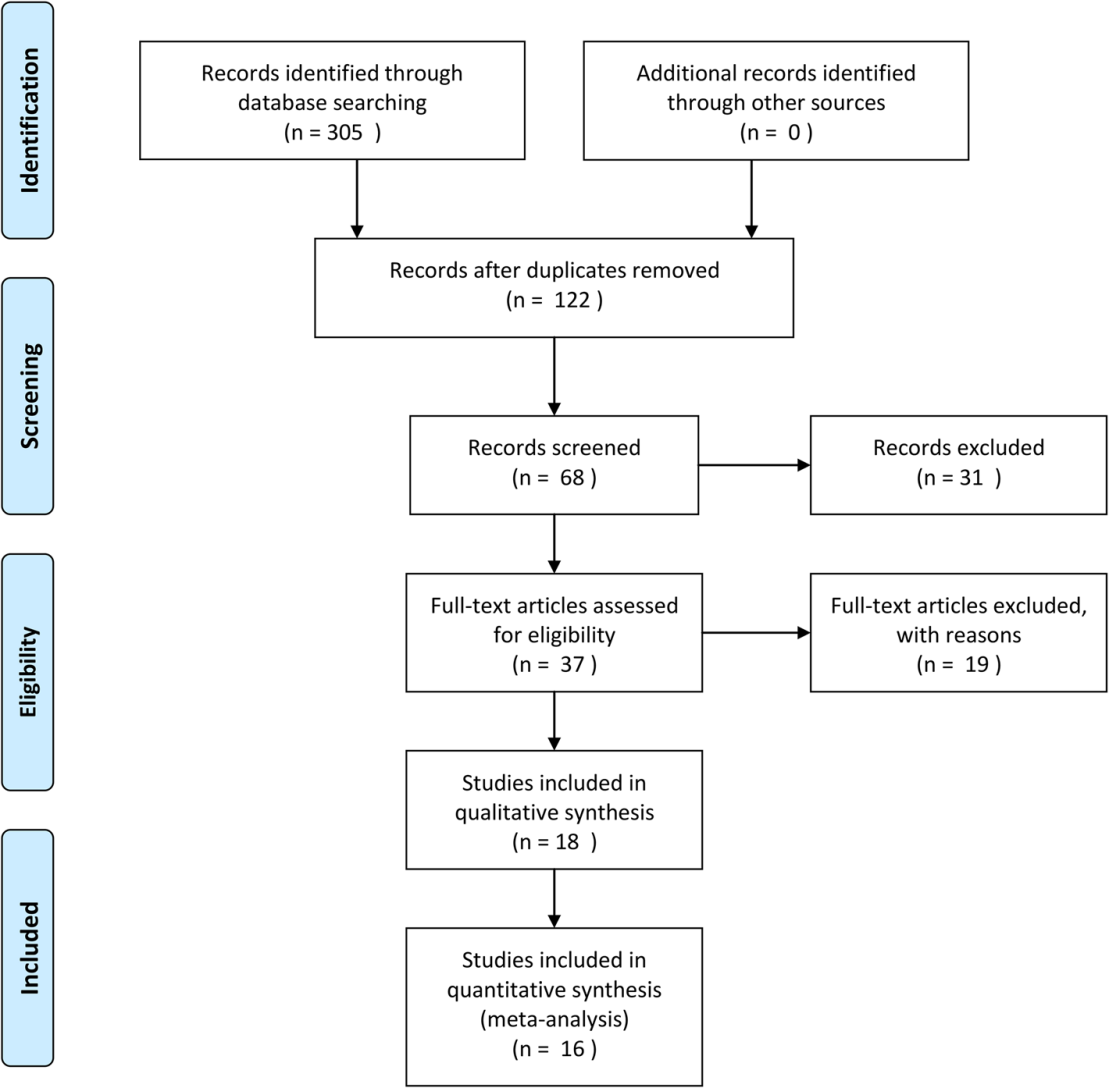
Flow diagram demonstrating inclusion/exclusion process for incorporate studies in final analyses

**Fig. 2:**
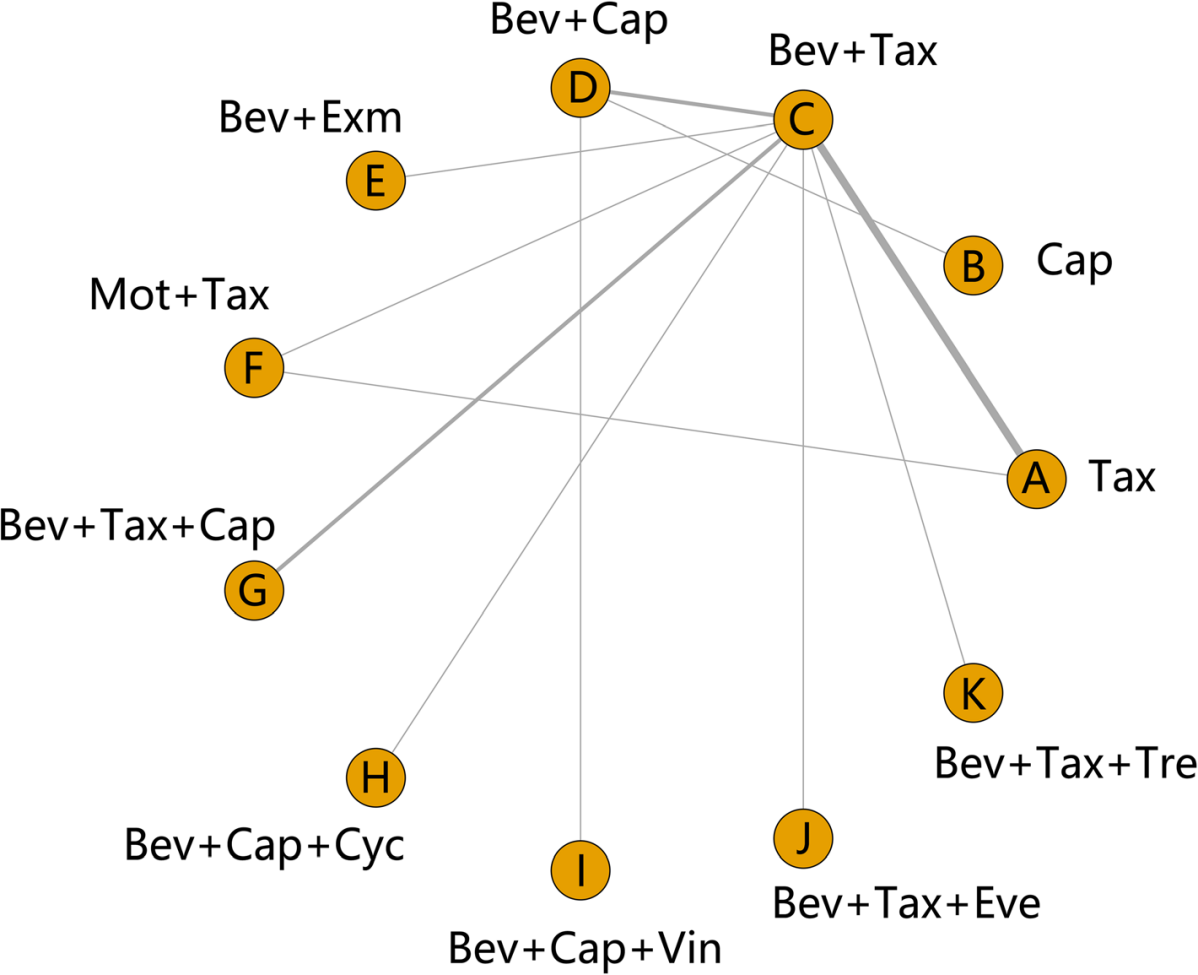
A network meta-analysis of interventional strategies for the treatment of metastatic breast cancer. Bev = bevacizumab, Cap = capecitabine, Vin = vinorelbine, Cyc = cyclophosphamide, Exm = exemestane, Eve = everolimus, Tre = trebananib, Mot = motesanib

The search identified 305 potentially relevant studies, of which 122 were included after duplicates removed. In total, 68 studies were retained for title and abstract review. By analyzing detail data, 37 studies were considered after full-text review. Moreover, 18 studies were included in qualitative synthesis, and two were duplicated data. Finally, sixteen studies were identified involving 589 patients that fulfilled the inclusion criteria in Fig. 1 [7–22]. Figure 2 demonstrates all available direct comparisons across outcomes in this network meta-analysis.

### Characteristics and methodological quality of the included studies

According to the PICOS principle (including “P” = patients, “I” = intervention, “C” = control, “O” = outcome, “S” = style), we presented the basic feature descriptions of the sixteen studies in Table 1. The age of enrolled patients arranged from 23 to 90 years. In hormone receptor status, the majority of HER2-negtive MBC patients were estrogen receptor (ER) positive and / or progesterone receptor (PR) positive, but the minority is patients with triple negative breast cancer. Moreover, more than half of the enrolled patients had received prior chemotherapy, while more than half of the patients with ER positive and / or PR positive had received prior hormonal therapy. Outcomes of all studies included PFS and ORR. All including studies were RCTs with a total of 5689 patients, which include one 3-arm trial and sixteen 2-arm trials. Eleven treatments, including Tax, Cap, Bev + Tax, Bev + Cap, Bev + Exm, Mot + Tax, Bev + Tax+Cap, Bev + Cap+Cyc, Bev + Cap+Vin, Bev + Tax+Eve, Bev + Tax+Tre, were involved in patients with HER2-negative metastatic breast cancer (Table 1).

**Table 1:** Characteristics of the studies included in the network meta-analysis

Study ID	Treatment 1								Treatment 2								Style
	n	Age (years)		Intervention	Receptor status		Prior therapy		n	Age (years)		Intervention	Receptor status		Prior therapy		
		Median	Range		ER+/PR+	TN	Chemo	Hormono		Median	Range		ER+/PR+	TN	Chemo	Hormono	
Norikazu Masuda 2017 [7]	24	53	32–78	Bev + Tax	22	2	9	15	30	60	28–71	Tax	24	6	13	19	RCT
David Miles 2017 [8]	239	55	28–85	Bev + Tax	200	39	116	92	242	56	28–77	Tax	203	39	118	105	RCT
Christoph Rochlitz 2016 [19]	71	64	30–82	Bev + Tax	61	10	38	NA	68	62	29–81	Bev + Cap+Cyc	52	15	37	NA	RCT
Christoph Zielinski 2016 [12]	266	NA	NA	Bev + Tax	205	60	142	50	265	NA	NA	Bev + Cap	201	64	143	43	RCT
A. Welt 2016 [16]	295	63	34–88	Bev + Cap+Vin	233	61	195	169	297	61	29–85	Bev + Cap	236	61	193	171	RCT
O. Trédan 2016 [20]	58	56	35–77	Bev + Exm	57	1	35	33	59	55	35–86	Bev + Tax	59	0	38	39	RCT
Denise A. Yardley 2015 [17]	56	61	30–77	Bev + Tax+Exr	44	12	34	34	57	57	25–79	Bev + Tax	45	12	31	37	RCT
Veronique Dieras 2015 [18]	56	57	32–75	Bev + Tax+Tre	45	9	10	NA	58	52	31–74	Bev + Tax	45	12	11	NA	RCT
Hans-Joachim Luck 2015 [15]	111	57	31–78	Bev + Tax+Cap	111	0	NA	NA	116	57	31–80	Bev + Tax	116	0	NA	NA	RCT
S.W. Lam 2014 [13]	156	56	32–76	Bev + Tax+Cap	133	23	90	82	156	56	34–74	Bev + Tax	132	24	89	76	RCT
Joseph Gligorov 2014 [14]	91	49	24–80	Bev + Tax+Cap	66	25	NA	NA	94	54	24–77	Bev + Tax	73	21	NA	NA	RCT
Istvan Lang 2013 [11]	279	59	48–65	Bev + Cap	212	67	176	171	285	59	49–64	Bev + Tax	222	63	180	175	RCT
Adam M. Brufsky 2011 [21]	201	56	28–86	Bev + Tax	145	47	NA	NA	103	56	35–84	Tax	77	20	NA	NA	RCT
	97	57	31–78	Bev + Cap	74	18	NA	NA	47	50	23–90	Cap	36	10	NA	NA	RCT
Miguel Martin 2011 [22]	97	55	44–66	Bev + Tax	78	19	64	64	94	53	43–63	Tax	75	19	62	56	RCT
	91	55	44–66	Mot + Tax	73	18	60	58	94	53	43–63	Tax	75	19	62	56	RCT
	97	55	44–66	Bev + Tax	78	19	64	64	91	55	44–66	Mot + Tax	73	18	60	58	RCT
David W. Miles 2010 [9]	247	55	27–76	Bev + Tax	187	60	167	120	241	55	29–83	Tax	189	52	156	135	RCT
Robert Gray 2009 [10]	368	56	29–84	Bev + Tax	223	120	244	NA	354	55	27–85	Tax	223	99	231	NA	RCT

*Bev* bevacizumab, *Cap* capecitabine, *Vin* vinorelbine, *Cyc* cyclophosphamide, *Exm* exemestane, *Exr* everolimus, *Tre* trebananib, *Mot* motesanib, *RCTs* randomized controlled trials, *NA* not applicable, *n* number of patients, *Receptor Status* hormone receptor status, *ER+/PR+* estrogen receptor (ER) and/or progesterone receptor (PR) positive, *TN* triple negative, *Chemo* prior chemotherapy, *Hormono* prior hormonal therapy

For the sixteen included studies, two investigators independently collected data and assessed methodological quality using the Cochrane collaboration’s tool for assessing risk of bias. Remarkably, most assessment items have high/moderate levels of methodological quality in this network meta-analysis (“A” and “B” level on the risk of bias), which results are shown in Table 2.

**Table 2:** Internal validity of included studies

Study	Prospective design	Multicenter enrollment	Selection bias	Performance bias	Attrition bias	Detection bias	Multivariate adjustment for potential confounders
Norikazu Masuda 2017 [7]	●	○	B	C	B	C	none reported
David Miles 2017 [8]	●	○	A	A	B	A	probably adequate
Christoph Rochlitz 2016 [20]	●	●	B	B	B	C	none reported
Christoph Zielinski 2016 [13]	●	●	A	A	B	A	probably adequate
A. Welt 2016 [17]	●	●	A	A	A	A	probably adequate
O. Trédan 2016 [21]	●	●	B	C	B	C	none reported
Denise A. Yardley 2015 [18]	●	○	B	C	A	C	none reported
Veronique Dieras 2015 [19]	●	●	B	C	A	C	none reported
Hans-Joachim Luck 2015 [16]	●	○	A	A	A	B	probably adequate
S.W. Lam 2014 [14]	●	●	A	A	A	B	probably adequate
Joseph Gligorov 2014 [15]	●	●	A	A	B	B	probably adequate
Istvan Lang 2013 [12]	●	●	A	A	B	A	probably adequate
Adam M. Brufsky 2011 [22]	●	●	A	B	A	A	probably adequate
Miguel Martin 2011 [23]	●	●	A	B	A	B	probably adequate
David W. Miles 2010 [9]	●	●	A	A	A	A	probably adequate
Robert Gray 2009 [10]	●	●	A	A	A	A	probably adequate

● = yes, ○ = no. Risk of bias is expressed as A = low, B = moderate, C = high, or D = incomplete reporting

### Heterogeneity, consistency and publication bias analysis

**Fig. 3:**
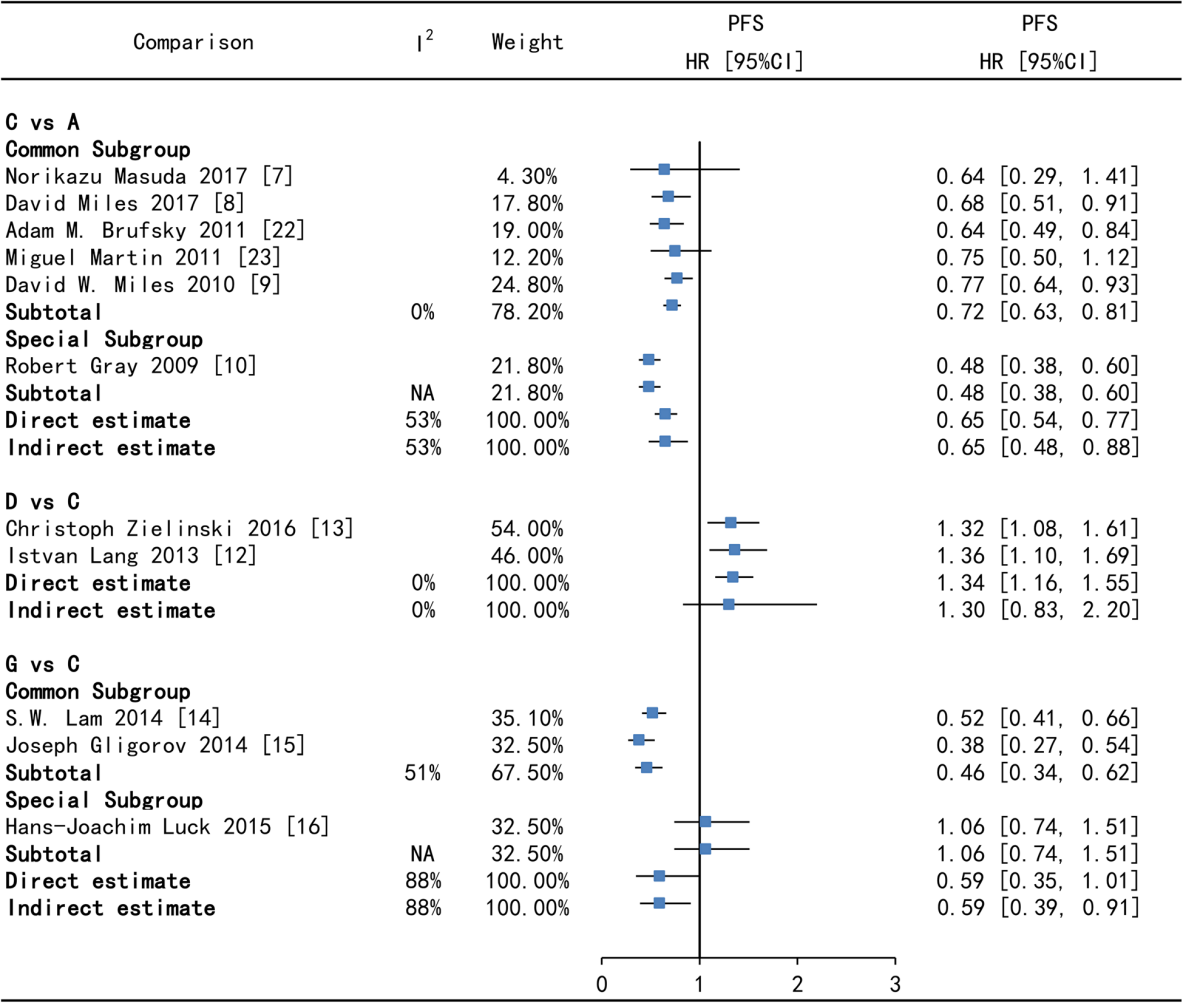
Forest plots of direct and indirect comparison for progression-free survival (PFS) - I. A = Tax, C = Bev + Tax, D = Bev + Cap, G = Bev + Tax+Cap. Bev = bevacizumab, Cap = capecitabine, Tax = taxanes. HR [95%CI] = hazard ratio with 95% confidence interval, NA = not applicable

Direct comparisons often suffered from limitations of risk of bias and imprecision, even heterogeneity after pooled. On PFS, Bev + Tax+Cap versus Bev + Tax has high heterogeneity (88%), however which reduce to moderate heterogeneity (51%) after subgroup analysis. Since one study show that Bev + Tax+Cap is not superior to Bev + Tax on PFS [15], which is contrary to the findings of two other studies [13, 14]. On ORR, Bev + Tax+Cap versus Bev + Tax has low heterogeneity (34%) in direct and indirect comparison, which may be because the ORR of Bev + Tax+Cap is higher than Bev + Tax, but close in one study [15]. The forest plot of direct and indirect comparison shows that Bev + Tax versus Tax has moderate heterogeneity (53%) on PFS and 47% on ORR. In subgroup analysis, there is no heterogeneity, except of one study which enrolled MBC not previously treated with chemotherapy [10]. The comparison of Bev + Cap versus Bev + Tax has no heterogeneity on PFS and ORR in Figs. 3 and 5.

**Fig. 4:**
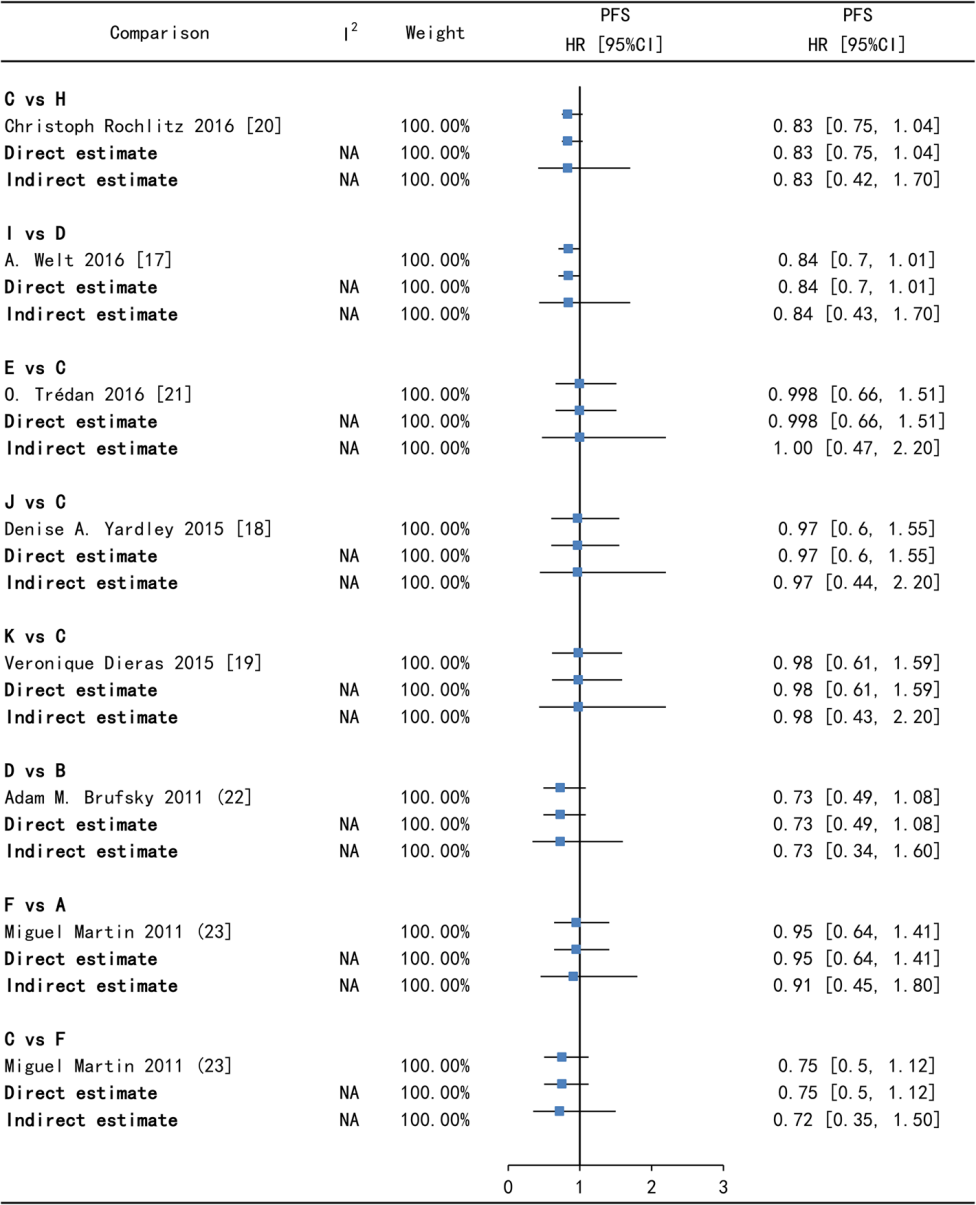
Forest plots of direct and indirect comparison for progression-free survival (PFS) - II. A = Tax, B = Cap, C = Bev + Tax, D = Bev + Cap, E = Bev + Exm, F = Mot + Tax, H = Bev + Cap+Cyc, I = Bev + Cap+Vin, J = Bev + Tax+ Eve, K = Bev + Tax+Tre. Bev = bevacizumab, Cap = capecitabine, Tax = taxanes, Vin = vinorelbine, Cyc = cyclophosphamide, Exm = exemestane, Eve = everolimus, Tre = trebananib, Mot = motesanib. HR [95%CI] = hazard ratio with 95% confidence interval, NA = not applicable

**Fig. 5:**
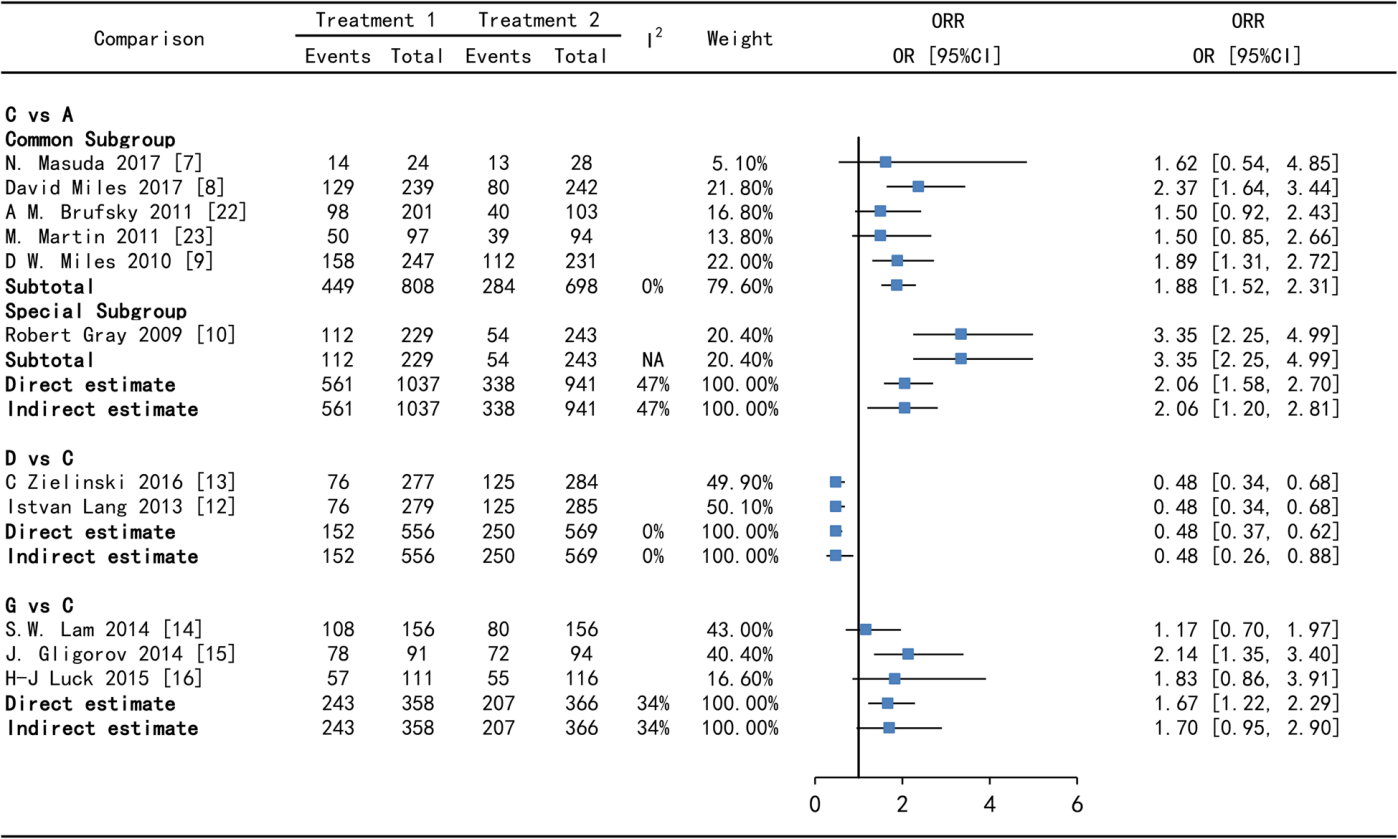
Forest plots of direct and indirect comparison for overall response rates (ORR) - I. A = Tax, C = Bev + Tax, D = Bev + Cap, G = Bev + Tax+Cap. Bev = bevacizumab, Cap = capecitabine, Tax = taxanes. OR [95%CI] = Odds ratio with 95% confidence interval, NA = not applicable

**Fig. 6:**
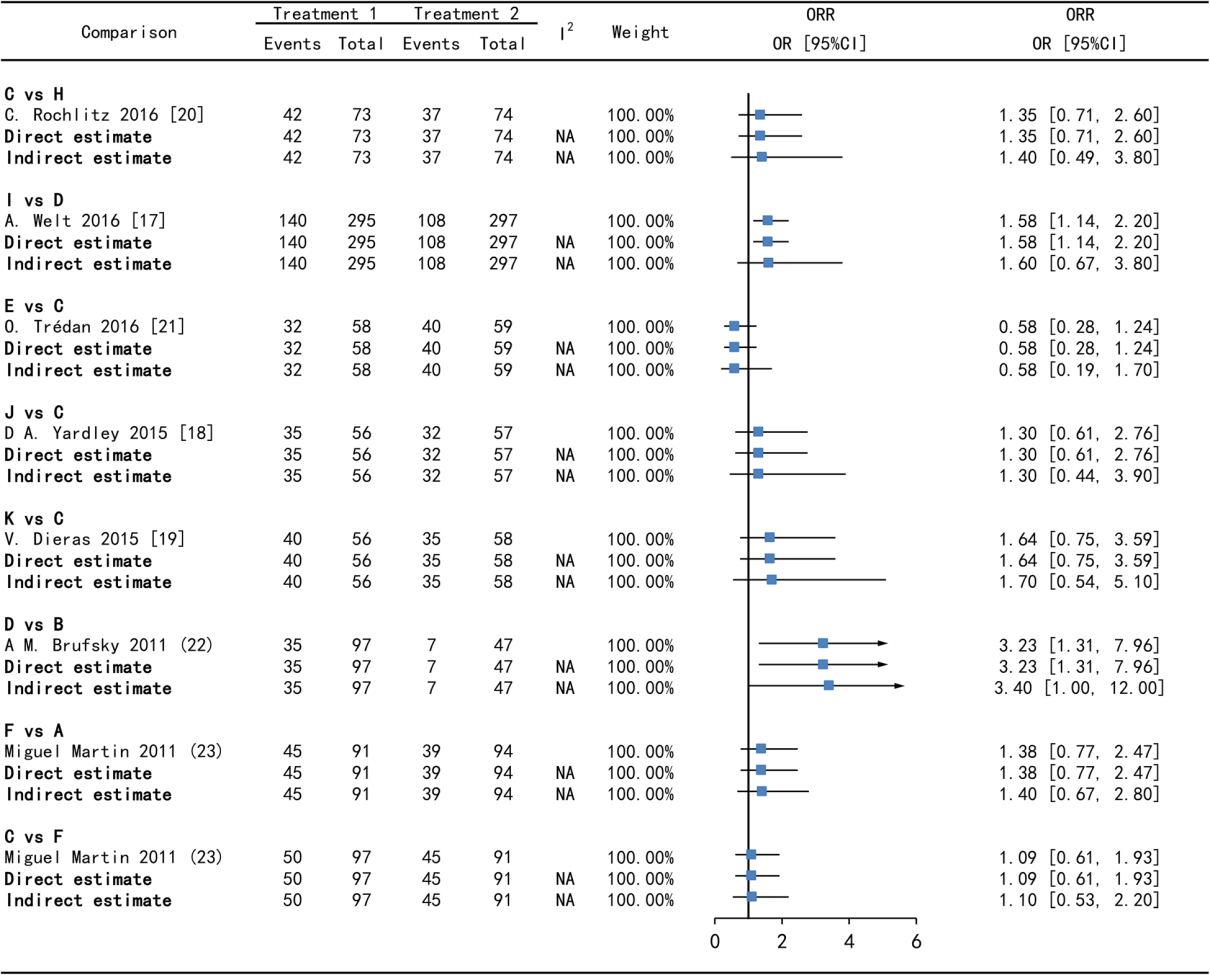
Forest plots of direct and indirect comparison for overall response rates (ORR) - II. A = Tax, B = Cap, C = Bev + Tax, D = Bev + Cap, E = Bev + Exm, F = Mot + Tax, H = Bev + Cap+Cyc, I = Bev + Cap+Vin, J = Bev + Tax+ Eve, K = Bev + Tax+Tre. Bev = bevacizumab, Cap = capecitabine, Tax = taxanes, Vin = vinorelbine, Cyc = cyclophosphamide, Exm = exemestane, Eve = everolimus, Tre = trebananib, Mot = motesanib. OR [95%CI] = Odds ratio with 95% confidence interval, NA = not applicable

For all comparisons across all outcomes, Node-splitting analysis suggested that there was no significantly consistency between direct and indirect estimates in Figs. 3, 4, 5 and 6. In Tax (A) - Bev + Tax (C) - Mot + Tax (F) closed loop, there is no significant difference on PFS and on ORR (the *p*-value of A versus C is 0.995775, A versus F is 0.997075 and C versus F is 0.993300) in Figs.3, 4, 5 and 6.

**Fig. 7:**
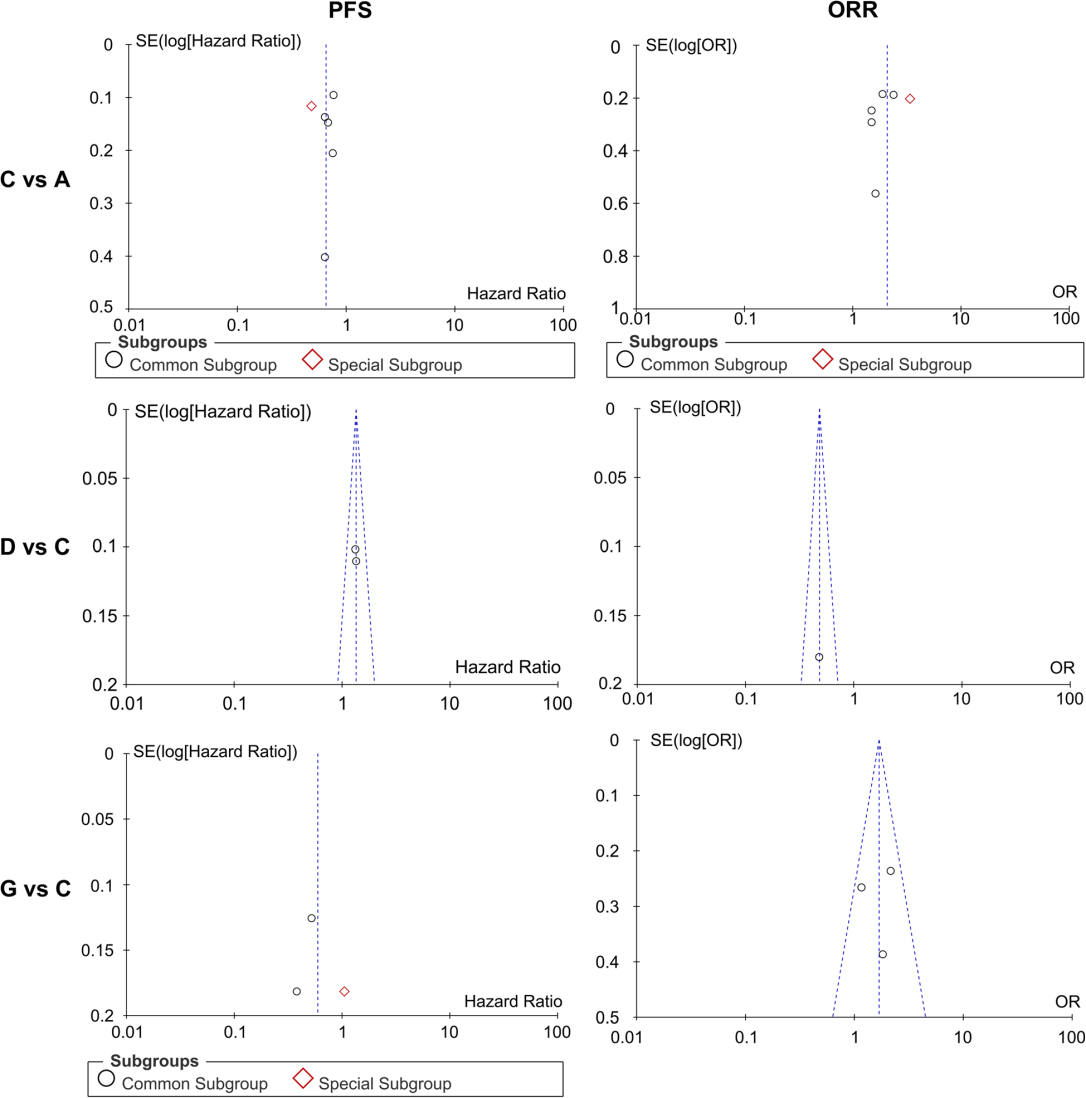
Funnel plots of the publication bias tests for direct comparisons of progression-free survival (PFS) and overall response rates (ORR). A = Tax, C = Bev + Tax, D = Bev + Cap, G = Bev + Tax+Cap. Bev = bevacizumab, Cap = capecitabine, Tax = taxanes

In addition, six direct comparisons, including Bev + Tax versus Tax, Bev + Cap versus Bev + Tax, Bev + Tax+Cap versus Bev + Tax on PFS and ORR were close to symmetric and no significant publication bias in Fig. 7.

### Progression-free survival

**Fig. 8:**
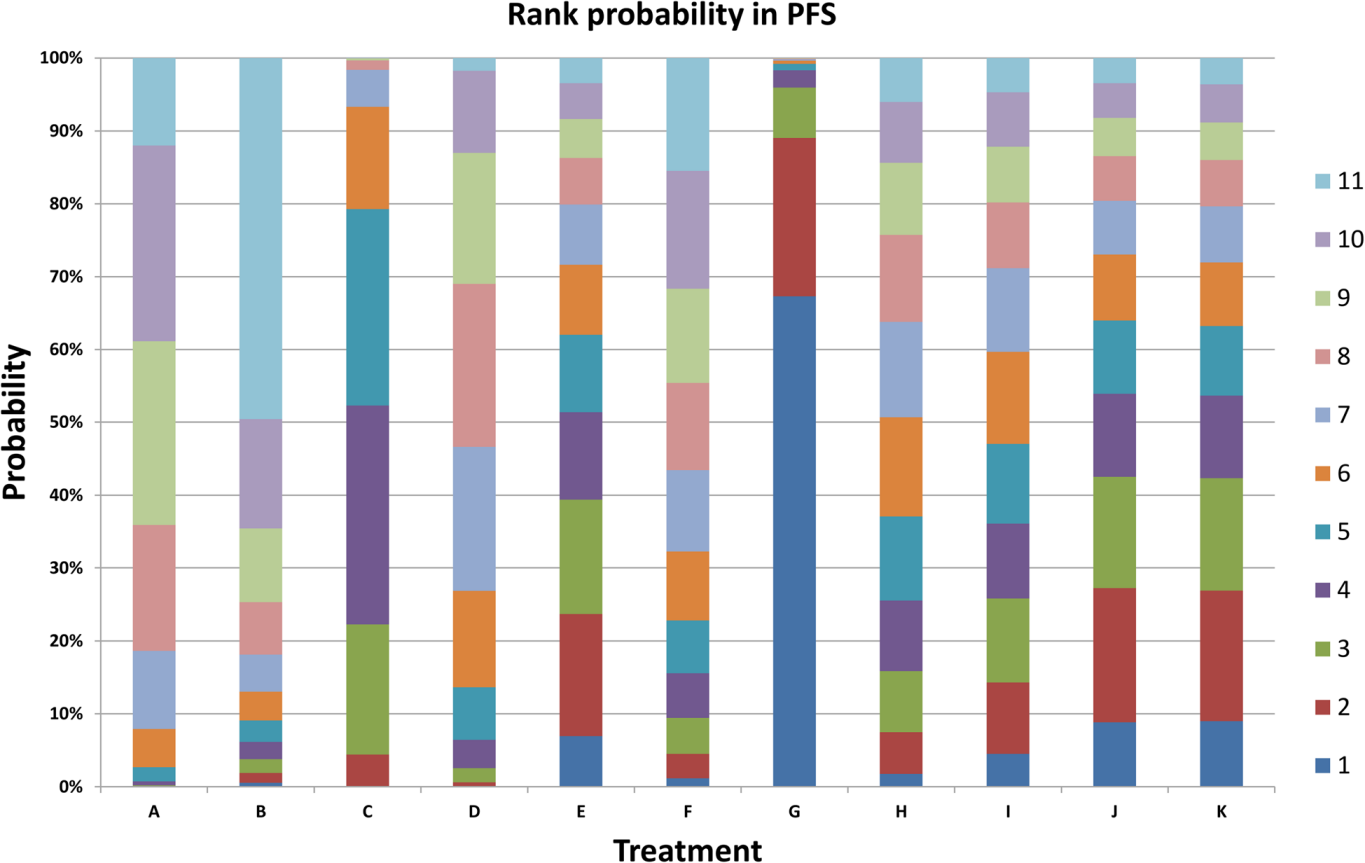
Cumulative ranking probability of progression-free survival (PFS) for the treatment of metastatic breast cancer. A = Tax, B = Cap, C = Bev + Tax, D = Bev + Cap, E = Bev + Exm, F = Mot + Tax, G = Bev + Tax+Cap, H = Bev + Cap+Cyc, I = Bev + Cap+Vin, J = Bev + Tax+Eve, K = Bev + Tax+Tre. Bev = bevacizumab, Cap = capecitabine, Tax = taxanes, Vin = vinorelbine, Cyc = cyclophosphamide, Exm = exemestane, Eve = everolimus, Tre = trebananib, Mot = motesanib. Serial number 1–11 represent probability ranking

Sixteen RCTs with 5689 patients reported on PFS. For six comparisons, the network estimate provided moderate-quality evidence with Bev + Tax versus Tax (HR = 0.65, 95%CI = 0.48–0.88), Bev + Tax+Cap versus Tax (HR = 0.38, 95%CI = 0.23–0.65), Bev + Tax+Cap versus Cap (HR = 0.32, 95%CI = 0.12–0.87), Bev + Tax+Cap versus Bev + Tax (HR = 0.59, 95%CI = 0.39–0.91), Bev + Tax+Cap versus Bev + Cap (HR = 0.44, 95%CI = 0.23–0.83), Bev + Tax+Cap versus Mot + Tax (HR = 0.42, 95%CI = 0.18–0.99). Other pairwise comparisons were not statistically significant difference (Table 3). The cumulative probability statistic showed that Bev + Tax+Cap ranked first, followed by Bev + Cap+Vin, Bev + Tax+Eve, Bev + Tax+Tre, Bev + Tax, Bev + Exm, Bev + Cap, Bev + Cap+Cyc, Mot + Tax, Tax and Cap. To reasonable evaluated the efficacy of bevacizumab-contained chemotherapy, the independent rank of bevacizumab combined with two chemotherapy agents is as flowing: Bev + Tax+Cap>Bev + Cap+Vin>Bev + Tax+Eve>Bev + Tax+Tre>Bev + Cap+Cyc; the rank of bevacizumab combined with chemotherapy agent: Bev + Tax>Bev + Exm>Bev + Cap (Fig. 8).

**Table 3:** Indirect comparison in PFS

**A**	1.2 (0.46, 3.1)	**0.65 (0.48, 0.88)**	0.87 (0.50, 1.5)	0.65 (0.29, 1.5)	0.91 (0.45, 1.8)	**0.38 (0.23, 0.65)**	0.78 (0.37, 1.6)	0.73 (0.30, 1.8)	0.63 (0.27, 1.5)	0.63 (0.27, 1.5)
0.84 (0.33, 2.2)	**B**	0.55 (0.22, 1.3)	0.73 (0.34, 1.6)	0.54 (0.17, 1.8)	0.76 (0.24, 2.4)	**0.32 (0.12, 0.87)**	0.66 (0.22, 2.0)	0.61 (0.22, 1.7)	0.53 (0.16, 1.7)	0.53 (0.16, 1.8)
**1.5 (1.1, 2.1)**	1.8 (0.75, 4.5)	**C**	1.3 (0.83, 2.2)	1.0 (0.47, 2.2)	1.4 (0.67, 2.8)	**0.59 (0.39, 0.91)**	1.2 (0.61, 2.4)	1.1 (0.49, 2.6)	0.97 (0.44, 2.2)	0.98 (0.43, 2.2)
1.1 (0.65, 2.0)	1.4 (0.64, 2.9)	0.75 (0.46, 1.2)	**D**	0.74 (0.31, 1.8)	1.0 (0.44, 2.5)	**0.44 (0.23, 0.83)**	0.90 (0.38, 2.0)	0.84 (0.43, 1.7)	0.73 (0.28, 1.9)	0.73 (0.28, 1.8)
1.5 (0.67, 3.4)	1.8 (0.57, 5.9)	1.0 (0.46, 2.1)	1.3 (0.54, 3.3)	**E**	1.4 (0.49, 4.0)	0.59 (0.24, 1.4)	1.2 (0.43, 3.3)	1.1 (0.36, 3.5)	0.97 (0.32, 3.0)	0.98 (0.32, 3.0)
1.1 (0.55, 2.2)	1.3 (0.42, 4.2)	0.72 (0.35, 1.5)	0.96 (0.41, 2.3)	0.71 (0.25, 2.0)	**F**	**0.42 (0.18, 0.99)**	0.86 (0.32, 2.3)	0.81 (0.27, 2.5)	0.70 (0.24, 2.1)	0.70 (0.24, 2.1)
**2.6 (1.5, 4.4)**	**3.1 (1.2, 8.3)**	**1.7 (1.1, 2.6)**	**2.3 (1.2, 4.3)**	1.7 (0.71, 4.1)	**2.4 (1.0, 5.4)**	**G**	2.1 (0.90, 4.5)	1.9 (0.75, 4.9)	1.7 (0.67, 4.0)	1.7 (0.66, 4.1)
1.3 (0.61, 2.7)	1.5 (0.50, 4.6)	0.83 (0.42, 1.7)	1.1 (0.49, 2.6)	0.83 (0.30, 2.3)	1.2 (0.43, 3.1)	0.49 (0.22, 1.1)	**H**	0.94 (0.32, 2.8)	0.81 (0.29, 2.3)	0.81 (0.28, 2.3)
1.4 (0.55, 3.3)	1.6 (0.59, 4.5)	0.89 (0.38, 2.0)	1.2 (0.60, 2.3)	0.88 (0.28, 2.8)	1.2 (0.41, 3.7)	0.52 (0.20, 1.3)	1.1 (0.36, 3.1)	**I**	0.86 (0.27, 2.7)	0.87 (0.26, 2.8)
1.6 (0.67, 3.7)	1.9 (0.58, 6.3)	1.0 (0.46, 2.3)	1.4 (0.54, 3.5)	1.0 (0.34, 3.1)	1.4 (0.48, 4.2)	0.61 (0.25, 1.5)	1.2 (0.43, 3.5)	1.2 (0.37, 3.6)	**J**	1.0 (0.32, 3.1)
1.6 (0.66, 3.7)	1.9 (0.57, 6.4)	1.0 (0.46, 2.3)	1.4 (0.55, 3.6)	1.0 (0.34, 3.1)	1.4 (0.48, 4.2)	0.60 (0.24, 1.5)	1.2 (0.43, 3.5)	1.2 (0.36, 3.8)	1.0 (0.32, 3.1)	**K**

A = Tax, B = Cap, C = Bev + Tax, D = Bev + Cap, E = Bev + Exm, F = Mot + Tax, G = Bev + Tax+Cap, H = Bev + Cap+Cyc, I = Bev + Cap+Vin, J = Bev + Tax+ Eve, K = Bev + Tax+Tre

The values represent HR (95%CI), and the values in bold represent HR (95%CI) has significant statistical difference in indirect comparison

*Bev* bevacizumab, *Cap* capecitabine, *Tax* taxanes, *Vin* vinorelbine, *Cyc* cyclophosphamide, *Exm* exemestane, *Eve* everolimus, *Tre* trebananib, *Mot* motesanib

### Objective response rate

**Fig. 9:**
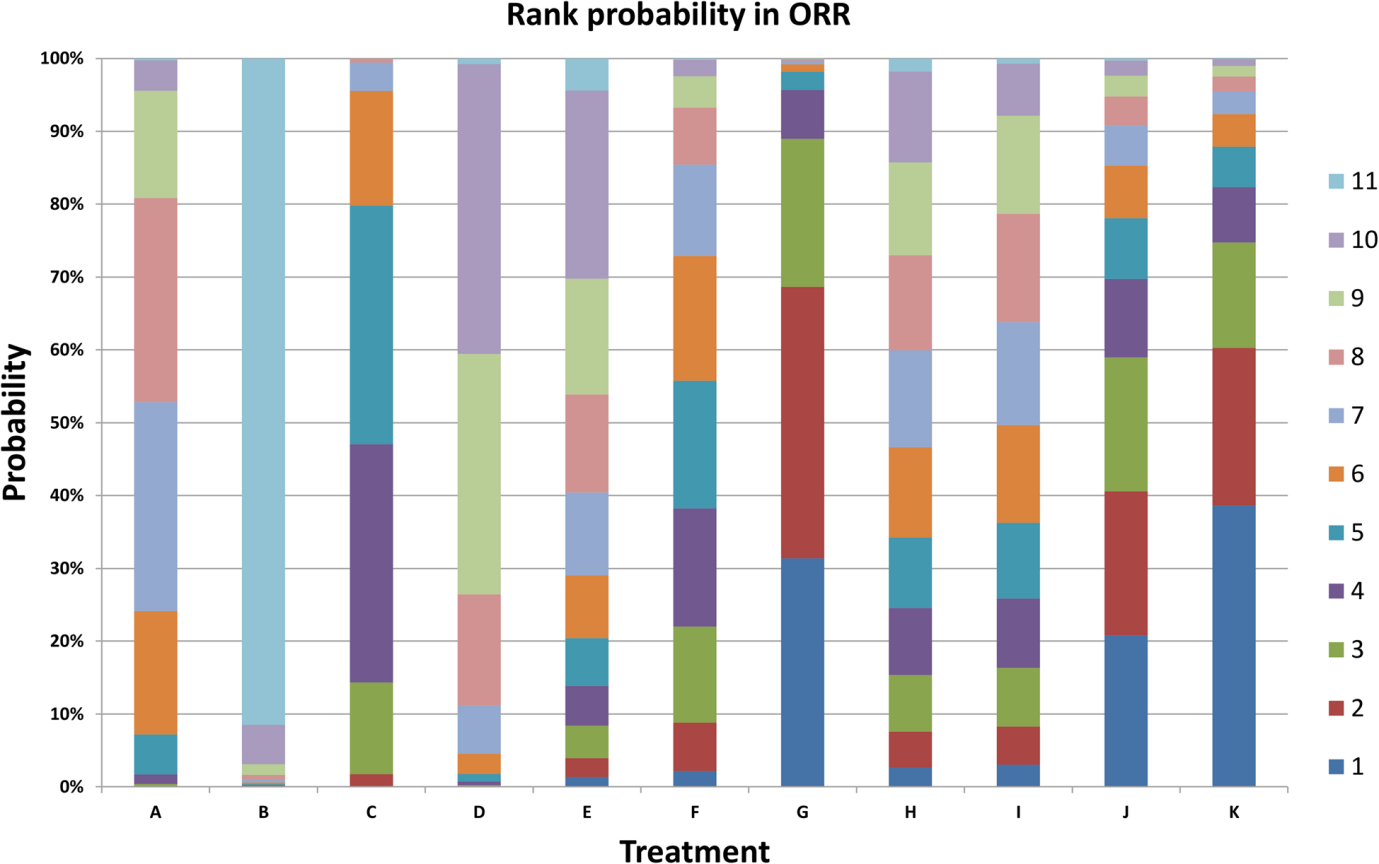
Cumulative ranking probability of overall response rate (ORR) for the treatment of metastatic breast cancer. A = Tax, B = Cap, C = Bev + Tax, D = Bev + Cap, E = Bev + Exm, F = Mot + Tax, G = Bev + Tax+Cap, H = Bev + Cap+Cyc, I = Bev + Cap+Vin, J = Bev + Tax+Eve, K = Bev + Tax+Tre. Bev = bevacizumab, Cap = capecitabine, Tax = taxanes, Vin = vinorelbine, Cyc = cyclophosphamide, Exm = exemestane, Eve = everolimus, Tre = trebananib, Mot = motesanib. Serial number 1–11 represent probability ranking

For objective response rate, sixteen studies (5689 patients) proved eligible. The results provide moderate quality evidence that Cap versus Tax (OR = 0.21, 95%CI = 0.051–0.85), Bev + Tax+Cap versus Tax (OR = 2.5, 95%CI = 1.3–4.9), Bev + Tax versus Cap (OR = 7.1, 95%CI = 1.9–28.0), Bev + Tax versus Tax (OR = 2.06, 95%CI = 1.20–2.81), Mot + Tax versus Cap (OR = 6.5, 95%CI = 1.4–31.0), Bev + Tax+Cap versus Cap (OR = 12, 95%CI = 2.8–52.0), Bev + Cap+Vin versus Cap (OR = 5.4, 95%CI = 1.3–24.0), Bev + Tax+Eve versus Cap (OR = 9.3, 95%CI = 1.7–53.0), Bev + Tax+Tre versus Cap (OR = 12, 95%CI = 2.1–69.0), Bev + Cap versus Bev + Tax (OR = 0.48, 95%CI = 0.26–0.88), Bev + Tax+Cap versus Bev + Cap (OR = 3.5, 95%CI = 1.5–8.0), Bev + Cap versus Cap (OR = 0.3, 95%CI = 0.085–0.96) and other pairwise comparisons were not statistically significant difference in Table 4. The therapeutic strategies ranking: Bev + Tax+Tre, Bev + Tax+Cap, Bev + Tax+Eve, Bev + Tax, Mot + Tax, Bev + Cap+Vin, Bev + Cap+Cyc, Tax, Bev + Cap, Bev + Exm, and Cap. Moreover, the independent rank of bevacizumab combined with two chemotherapy agents: Bev + Tax+Tre>Bev + Tax+Cap>Bev + Tax+Eve>Bev + Cap+Vin>Bev + Cap+Cyc; the rank of bevacizumab combined with chemotherapy agent: Bev + Tax>Bev + Cap>Bev + Exm (Fig. 9).

**Table 4:** Indirect comparison in ORR

**A**	**0.21 (0.051, 0.85)**	**2.06 (1.20, 2.81)**	0.72 (0.35, 1.5)	0.87 (0.28, 2.8)	1.4 (0.67, 2.8)	**2.5 (1.3, 4.9)**	1.1 (0.37, 3.3)	1.1 (0.37, 3.5)	2.0 (0.62, 6.2)	2.5 (0.77, 8.2)
**4.7 (1.2, 20.0)**	**B**	**7.1 (1.9, 28.0)**	**3.4 (1.0, 12.0)**	4.1 (0.71, 24.0)	**6.5 (1.4, 31.0)**	**12.0 (2.8, 52.0)**	5.2 (0.96, 29.0)	**5.4 (1.3, 24.0)**	**9.3 (1.7, 53.0)**	**12.0 (2.1, 69.0)**
**0.49 (0.36, 0.83)**	**0.14 (0.036, 0.53)**	**C**	**0.48 (0.26, 0.88)**	0.58 (0.19, 1.7)	0.92 (0.45, 1.9)	1.7 (0.95, 2.9)	0.73 (0.26, 2.0)	0.76 (0.26, 2.2)	1.3 (0.44, 3.9)	1.7 (0.54, 5.1)
1.4 (0.68, 2.8)	**0.30 (0.085, 0.96)**	**2.1 (1.1, 3.9)**	**D**	1.2 (0.34, 4.2)	1.9 (0.74, 4.9)	**3.5 (1.5, 8.0)**	1.5 (0.46, 5.1)	1.6 (0.67, 3.8)	2.7 (0.78, 9.7)	3.5 (0.96, 12.0)
1.2 (0.36, 3.6)	0.24 (0.042, 1.4)	1.7 (0.58, 5.2)	0.82 (0.24, 2.9)	**E**	1.6 (0.42, 5.9)	2.9 (0.83, 9.9)	1.3 (0.28, 5.6)	1.3 (0.28, 6.1)	2.3 (0.48, 11.0)	2.8 (0.60, 14.0)
0.72 (0.35, 1.5)	**0.15 (0.032, 0.70)**	1.1 (0.53, 2.2)	0.52 (0.20, 1.3)	0.63 (0.17, 2.4)	**F**	1.8 (0.73, 4.6)	0.80 (0.23, 2.8)	0.82 (0.23, 3.0)	1.4 (0.39, 5.2)	1.8 (0.48, 6.8)
**0.40 (0.20, 0.78)**	**0.085 (0.019, 0.36)**	0.60 (0.34, 1.1)	**0.29 (0.12, 0.66)**	0.35 (0.10, 1.2)	0.56 (0.22, 1.4)	**G**	0.44 (0.14, 1.4)	0.46 (0.14, 1.5)	0.79 (0.23, 2.7)	0.99 (0.28, 3.5)
0.91 (0.30, 2.7)	0.19 (0.034, 1.0)	1.4 (0.49, 3.8)	0.65 (0.20, 2.2)	0.79 (0.18, 3.5)	1.3 (0.36, 4.4)	2.3 (0.70, 7.4)	**H**	1.0 (0.24, 4.5)	1.8 (0.40, 8.1)	2.2 (0.49, 10.0)
0.88 (0.29, 2.7)	**0.19 (0.042, 0.80)**	1.3 (0.46, 3.8)	0.63 (0.27, 1.5)	0.77 (0.16, 3.5)	1.2 (0.34, 4.3)	2.2 (0.66, 7.3)	0.97 (0.22, 4.2)	**I**	1.7 (0.37, 8.0)	2.2 (0.47, 10.0)
0.51 (0.16, 1.6)	**0.11 (0.019, 0.61)**	0.76 (0.25, 2.3)	0.37 (0.10, 1.3)	0.44 (0.094, 2.1)	0.70 (0.19, 2.6)	1.3 (0.37, 4.4)	0.56 (0.12, 2.5)	0.58 (0.12, 2.7)	**J**	1.3 (0.26, 6.1)
0.40 (0.12, 1.3)	**0.085 (0.014, 0.48)**	0.60 (0.20, 1.8)	0.29 (0.080, 1.0)	0.35 (0.073, 1.7)	0.56 (0.15, 2.1)	1.0 (0.28, 3.5)	0.45 (0.097, 2.0)	0.46 (0.097, 2.1)	0.79 (0.16, 3.9)	**K**

A = Tax, B = Cap, C = Bev + Tax, D = Bev + Cap, E = Bev + Exm, F = Mot + Tax, G = Bev + Tax+Cap, H = Bev + Cap+Cyc, I = Bev + Cap+Vin, J = Bev + Tax+ Eve, K = Bev + Tax+Tre

The values represent OR (95%CI), and the values in bold represent OR (95%CI) has significant statistical difference in indirect comparison

*Bev* bevacizumab, *Cap* capecitabine, *Tax* taxanes, *Vin* vinorelbine, *Cyc* cyclophosphamide, *Exm* exemestane, *Eve* everolimus, *Tre* trebananib, *Mot* motesanib

### Safety

Summary frequency of treatment-related grade ≧ 3 adverse events (AE), including hematologic AE (anemia, leukopenia and neutropenia) and non-hematologic AE (hypertension, haemorrhage/bleeding, thromboembolic events, neuropathy, nausea/vomiting, diarrhea, mucositis/stomatitis, edema, proteinuria, hepatobiliary disorders, hand-foot syndrome, fatigue, pain, alopecia and infection) are pooled for analysis in Table 5. We found that the toxicity of regimens significantly increases with the addition of bevacizumab or chemotherapy drugs in general, even though the adverse events of Cap and Bev + Cap+Cyc regimens are not applicable.

**Table 5:** Grade ≥ 3 hematological and non-hematological adverse events

Toxicity	A		B		C		D		E		F		G		H		I		J		K	
(Grade ≥ 3)	Tax		Cap		Bev + Tax		Bev + Cap		Bev + Exm		Mot + Tax		Bev + Tax+Cap		Bev + Cap+Cyc		Bev + Cap+Vin		Bev + Tax+Exr		Bev + Tax+Tre	
Total n	351		47		1462		918		58		92		357		74		295		55		55	
	n_1_/n_2_	%	n_1_/n_2_	%	n_1_/n_2_	%	n_1_/n_2_	%	n_1_/n_2_	%	n_1_/n_2_	%	n_1_/n_2_	%	n_1_/n_2_	%	n_1_/n_2_	%	n_1_/n_2_	%	n_1_/n_2_	%
**Hematologic**			NA	NA											NA	NA						
Anemia	6/231	2.6			22/918	2.4	8/851	0.9	0/58	0.0			5/357	1.4					6/55	10.9		
Leukopenia	10/231	4.3			84/1145	7.3	3/851	0.4	0/58	0.0			37/357	10.4			32/295	10.8				
Neutropenia	37/262	14.1			267/1366	19.5	13/851	1.5	0/58	0.0			64/357	17.9			57/295	19.3	11/55	20.0	18/55	32.7
**Non-hematologic**			NA	NA											NA	NA						
Hypertension	11/351	3.1			133/1462	9.1	55/851	6.5	25/58	43.1	11/92	12.0	39/357	10.9			9/295	3.1	2/55	3.6	18/55	32.7
Haemorrhage/bleeding	2/262	0.8			7/624	1.1	3/297	1.0	0/58	0.0			5/246	2.0			3/295	1.0	1/55	1.8		
Thromboembolic events	3/262	1.1			17/742	2.3	18/297	6.1	1/58	1.7			28/357	7.8			31/295	10.5			5/55	9.1
Neuropathy	16/320	5.0			156/1448	10.8	3/851	0.4	0/58	0.0	10/92	10.9	19/357	5.3			11/295	3.7	6/55	10.9	20/55	36.4
Nausea/vomiting	7/320	2.2			28/956	2.9	12/574	2.1	0/58	0.0	18/92	19.6	8/155	5.2			19/295	6.4	3/55	5.5	4/55	7.3
Diarrhea	9/320	2.8			36/1164	3.1	29/574	5.1	0/58	0.0	3/92	3.3	21/357	5.9			10/295	3.4	3/55	5.5	4/55	7.3
Mucositis/stomatitis	2/320	0.6			25/763	3.3	8/297	2.7	0/58	0.0	1/92	1.1	20/357	5.6			8/295	2.7	8/55	14.5		
Edema	6/231	2.6			4/420	1.0			0/58	0.0											2/55	3.6
Proteinuria	1/262	0.4			14/565	2.5	3/297	1.0					18/246	7.3			1/295	0.3	4/55	7.3		
Hepatobiliary disorders	3/89	3.4			6/213	2.8			4/58	6.9	7/92	7.6									0/55	0.0
Hand-foot syndrome					4/648	0.6	157/851	18.4					107/357	30.0			43/295	14.6				
Fatigue	20/320	6.3			59/1164	5.1	10/574	1.7	3/58	5.2	11/92	12.0	22/357	6.2			19/295	6.4	8/55	14.5	5/55	9.1
Pain	11/320	3.4			22/989	2.2	9/277	3.2	2/58	3.4	9/92	9.8	4/246	1.6					1/55	1.8	0/55	0.0
Alopecia	9/320	2.8			13/685	1.9	0/277	0.0	0/58	0.0	0/92	0.0									4/55	7.3
Infection	10/320	3.1			19/615	3.1	27/297	9.1			9/92	9.8	16/266	6.0			37/295	12.5				

A = Tax, B = Cap, C = Bev + Tax, D = Bev + Cap, E = Bev + Exm, F = Mot + Tax, G = Bev + Tax+Cap, H = Bev + Cap+Cyc, I = Bev + Cap+Vin, J = Bev + Tax+ Eve, K = Bev + Tax+Tre

*Bev* bevacizumab, *Cap* capecitabine, *Tax* taxanes, *Vin* vinorelbine, *Cyc* cyclophosphamide, *Exm* exemestane, *Eve* everolimus, *Tre* trebananib, *Mot* motesanib, *NA* not applicable, *Total n* number of all patients with regimens, *n*_*1*_*/n*_*2*_ the number of patients with adverse reactions / total enrolled patients

## Discussion

In this network meta-analysis, we included 16 RCTs enrolling 5689 patients comparing various chemotherapy strategies. The use of indirect comparisons within this network meta-analysis adds additional information beyond the multiple direct comparison meta-analysis that have compared Bev + Tax, Bev + Cap, Bev + Tax+Cap with Tax, Cap and with other new chemotherapy. According to our results, it is certain that the addition of bevacizumab improved PFS and ORR compared with chemotherapy alone, which is consistent with previous studies [7, 8, 21]. Moreover, we found that more patients who received Bev + Tax had an objective response than did those who received Bev + Cap, and that Bev + Tax is superior to Bev + Cap in therapeutic strategies ranking, but there was no significant difference between Bev + Tax and Bev + Cap on PFS in HER2-negative breast cancer. Previous studies have also showed that progression-free survival with Bev + Tax is superior to that noted with Bev + Cap, but one of included RCTs has indicated that the advantage of Bev + Tax to Bev + Cap have not statistically difference on PFS [11, 12]. In addition, most included trials directly compared Bev + Tax with Tax, while few trials directly compared Bev + Cap with Cap and with Bev + Tax, which could impact our results in the indirect comparison of network meta-analysis.

The efficacy of bevacizumab combined with two chemotherapeutic agents was generally superior to bevacizumab combined with mono-chemotherapy on ORR, but there was no significant difference on PFS in patients with HER2-negative breast cancer [16–18]. In order to avoid the influence on the addition of second chemotherapy agent improves PFS and ORR compared with Bev + mono-chemotherapy alone in bevacizumab-containing regimens, the efficacy of bevacizumab combined with one or two chemotherapy agents has also been independent evaluated and ranked in this network meta-analysis. Of even greater concern is that Bev + Tax+Cap could be the best therapeutic strategy to improve PFS and ORR based on our currently evidences, which has highest-ranking in bevacizumab plus two chemotherapy agents, even the whole ranking. Besides, there were significant statistical differences compared with Bev + Cap or Bev + Tax or Cap or Tax, while several studies suggested that Bev + Tax+Cap significantly improved PFS and ORR, even have manageable tolerability, compared with Bev + Tax as first-line treatment [13, 14]. However, Bev + Tax+Cap cannot be recommended as first-line chemotherapy in a phase III study, while there was no significant difference between Bev + Tax+Cap and Bev + Tax [15]. In addition, We found that two antiangiogenic agents, bevacizumab and trebananib, combined with taxanes is great potential chemotherapy strategy in our independent ranking results of bevacizumab plus two chemotherapy agents, but only the comparisons of Bev + Tax+Tre and Cap have statistical differences in HER2-negative breast cancer. Based on available evidence, Bev + Cap+Cyc might not even be a better therapeutic regimen compared with bevacizumab plus mono-chemotherapy, which is consistent with the result of previous study [19]. Also of concern, the toxicity of therapeutic drugs could inevitably increase with multidrug treatment regimens in our pooled analysis of treatment-related grade ≧ 3 adverse events, thus it is necessary that finding a balance between the efficacy and toxicity when we choose appropriate therapeutic regimens.

Several limitations of our study deserve comment. First, the included RCTs on second-line chemotherapeutic agents (such as exemestane, everolimus, trebananib and motesanib) may not be sufficient, which caused the bias of our finding. Second, overall survival (OS) was not applicable to include and evaluate the efficacy of bevacizumab-containing chemotherapy regimens in this network meta-analysis. Third, we found that the cause of heterogeneity maybe the baseline of eligible patients in direct comparison, including MBC not previously treated with chemotherapy. However, hormone receptor status may also influent on the heterogeneity and which need to be further confirmed. And previous study suggested that bevacizumab-containing regimens are superior to chemotherapy alone on pathological complete response (pCR) in triple-negative breast cancer (TNBC), which maybe different than non-TNBC [23]. Fourth, due to the inconsistencies of adverse events among the included studies, it is hard to more accurate evaluate the safety of therapeutic regimens for meta-analysis in patients with HER2-negative metastatic breast cancer.

## Conclusions

In summary, our network meta-analysis results showed that Bev + Tax+Cap maybe the best therapeutic regimen on PFS and ORR, which was superior to bevacizumab combined with other chemotherapy drugs in HER2-negative metastatic breast cancer. However it should be also considered that bevacizumab may add toxicity to chemotherapy and whether improve overall survival (OS) or not.

## Data Availability

All data generated or analyzed during this study are included in this published article.

## Abbreviations

Bev: Bevacizumab
Cap: Capecitabine
CI: Confidence interval
Cyc: Cyclophosphamide
Eve: Everolimus
Exm: Exemestane
MBC: Metastatic breast cancer
Mot: Motesanib
pCR: Pathological complete response
RCTs: Randomized controlled trials
Tax: Taxanes
TNBC: Triple-negative breast cancer
Tre: Trebananib
Vin: Vinorelbine

## References

[1] Folkman J (1971). Tumor angiogenesis: therapeutic implications. N Engl J Med.

[2] Marty M, Pivot X (2008). The potential of anti-vascular endothelial growth factor therapy in metastatic breast cancer: clinical experience with anti-angiogenic agents, focusing on bevacizumab. Eur J Cancer.

[3] Brown LF, Berse B, Jackman RW, Tognazzi K, Guidi AJ, Dvorak HF, Senger DR, Connolly JL, Schnitt SJ (1995). Expression of vascular permeability factor (vascular endothelial growth factor) and its receptors in breast cancer. Hum Pathol.

[4] Ellis LM, Hicklin DJ (2008). VEGF-targeted therapy: mechanisms of anti-tumour activity. Nat Rev Cancer.

[5] Bear HD, Tang G, Rastogi P, Geyer CE, Robidoux A, Atkins JN, Baez-Diaz L, Brufsky AM, Mehta RS, Fehrenbacher L (2012). Bevacizumab added to neoadjuvant chemotherapy for breast cancer. N Engl J Med.

[6] von Minckwitz G, Eidtmann H, Rezai M, Fasching PA, Tesch H, Eggemann H, Schrader I, Kittel K, Hanusch C, Kreienberg R (2012). Neoadjuvant chemotherapy and bevacizumab for HER2-negative breast cancer. N Engl J Med.

[7] Masuda N, Takahashi M, Nakagami K, Okumura Y, Nakayama T, Sato N, Kanatani K, Tajima K, Kashiwaba M (2017). First-line bevacizumab plus paclitaxel in Japanese patients with HER2-negative metastatic breast cancer: subgroup results from the randomized Phase III MERiDiAN trial. Jpn J Clin Oncol.

[8] Miles D, Cameron D, Bondarenko I, Manzyuk L, Alcedo JC, Lopez RI, Im SA, Canon JL, Shparyk Y, Yardley DA (2017). Bevacizumab plus paclitaxel versus placebo plus paclitaxel as first-line therapy for HER2-negative metastatic breast cancer (MERiDiAN): a double-blind placebo-controlled randomised phase III trial with prospective biomarker evaluation. Eur J Cancer.

[9] Miles DW, Chan A, Dirix LY, Cortes J, Pivot X, Tomczak P, Delozier T, Sohn JH, Provencher L, Puglisi F (2010). Phase III study of bevacizumab plus docetaxel compared with placebo plus docetaxel for the first-line treatment of human epidermal growth factor receptor 2-negative metastatic breast cancer. J Clin Oncol.

[10] Gray R, Bhattacharya S, Bowden C, Miller K, Comis RL (2009). Independent review of E2100: a phase III trial of bevacizumab plus paclitaxel versus paclitaxel in women with metastatic breast cancer. J Clin Oncol.

[11] Lang I, Brodowicz T, Ryvo L, Kahan Z, Greil R, Beslija S, Stemmer SM, Kaufman B, Zvirbule Z, Steger GG (2013). Bevacizumab plus paclitaxel versus bevacizumab plus capecitabine as first-line treatment for HER2-negative metastatic breast cancer: interim efficacy results of the randomised, open-label, non-inferiority, phase 3 TURANDOT trial. Lancet Oncol.

[12] Zielinski C, Lang I, Inbar M, Kahan Z, Greil R, Beslija S, Stemmer SM, Zvirbule Z, Steger GG, Melichar B (2016). Bevacizumab plus paclitaxel versus bevacizumab plus capecitabine as first-line treatment for HER2-negative metastatic breast cancer (TURANDOT): primary endpoint results of a randomised, open-label, non-inferiority, phase 3 trial. Lancet Oncol.

[13] Lam SW, de Groot SM, Honkoop AH, Jager A, ten Tije AJ, Bos MM, Linn SC, van den Bosch J, Kroep JR, Braun JJ (2014). Paclitaxel and bevacizumab with or without capecitabine as first-line treatment for HER2-negative locally recurrent or metastatic breast cancer: a multicentre, open-label, randomised phase 2 trial. Eur J Cancer.

[14] Gligorov J, Doval D, Bines J, Alba E, Cortes P, Pierga JY, Gupta V, Costa R, Srock S, de Ducla S (2014). Maintenance capecitabine and bevacizumab versus bevacizumab alone after initial first-line bevacizumab and docetaxel for patients with HER2-negative metastatic breast cancer (IMELDA): a randomised, open-label, phase 3 trial. Lancet Oncol.

[15] Luck HJ, Lubbe K, Reinisch M, Maass N, Feisel-Schwickardi G, Tome O, Janni W, Aydogdu M, Neunhoffer T, Ober A (2015). Phase III study on efficacy of taxanes plus bevacizumab with or without capecitabine as first-line chemotherapy in metastatic breast cancer. Breast Cancer Res Treat.

[16] Welt A, Marschner N, Lerchenmueller C, Decker T, Steffens CC, Koehler A, Depenbusch R, Busies S, Hegewisch-Becker S (2016). Capecitabine and bevacizumab with or without vinorelbine in first-line treatment of HER2/neu-negative metastatic or locally advanced breast cancer: final efficacy and safety data of the randomised, open-label superiority phase 3 CARIN trial. Breast Cancer Res Treat.

[17] Yardley DA, Bosserman LD, O'Shaughnessy JA, Harwin WN, Morgan SK, Priego VM, Peacock NW, Bass JD, Burris HA, Hainsworth JD (2015). Paclitaxel, bevacizumab, and everolimus/placebo as first-line treatment for patients with metastatic HER2-negative breast cancer: a randomized placebo-controlled phase II trial of the Sarah Cannon Research Institute. Breast Cancer Res Treat.

[18] Dieras V, Wildiers H, Jassem J, Dirix LY, Guastalla JP, Bono P, Hurvitz SA, Goncalves A, Romieu G, Limentani SA (2015). Trebananib (AMG 386) plus weekly paclitaxel with or without bevacizumab as first-line therapy for HER2-negative locally recurrent or metastatic breast cancer: a phase 2 randomized study. Breast.

[19] Rochlitz C, Bigler M, von Moos R, Bernhard J, Matter-Walstra K, Wicki A, Zaman K, Anchisi S, Kung M, Na KJ (2016). SAKK 24/09: safety and tolerability of bevacizumab plus paclitaxel vs. bevacizumab plus metronomic cyclophosphamide and capecitabine as first-line therapy in patients with HER2-negative advanced stage breast cancer - a multicenter, randomized phase III trial. BMC Cancer.

[20] Tredan O, Follana P, Moullet I, Cropet C, Trager-Maury S, Dauba J, Lavau-Denes S, Dieras V, Beal-Ardisson D, Gouttebel M (2016). A phase III trial of exemestane plus bevacizumab maintenance therapy in patients with metastatic breast cancer after first-line taxane and bevacizumab: a GINECO group study. Ann Oncol.

[21] Brufsky AM, Hurvitz S, Perez E, Swamy R, Valero V, O'Neill V, Rugo HS (2011). RIBBON-2: a randomized, double-blind, placebo-controlled, phase III trial evaluating the efficacy and safety of bevacizumab in combination with chemotherapy for second-line treatment of human epidermal growth factor receptor 2-negative metastatic breast cancer. J Clin Oncol.

[22] Martin M, Roche H, Pinter T, Crown J, Kennedy MJ, Provencher L, Priou F, Eiermann W, Adrover E, Lang I (2011). Motesanib, or open-label bevacizumab, in combination with paclitaxel, as first-line treatment for HER2-negative locally recurrent or metastatic breast cancer: a phase 2, randomised, double-blind, placebo-controlled study. Lancet Oncol.

[23] Li Y, Yang D, Chen P, Yin X, Sun J, Li H, Ren G (2019). Efficacy and safety of neoadjuvant chemotherapy regimens for triple-negative breast cancer: a network meta-analysis. Aging (Albany NY).

